# Academic productivism: when job demand exceeds working time

**DOI:** 10.11606/s1518-8787.2020054002288

**Published:** 2020-11-17

**Authors:** Talita da Silveira Campos Teixeira, Elaine Cristina Marqueze, Claudia Roberta de Castro Moreno

**Affiliations:** I Universidade de São Paulo Faculdade de Saúde Pública São PauloSP Brasil Universidade de São Paulo. Faculdade de Saúde Pública. Programa de Pós-Graduação em Saúde Pública. São Paulo, SP, Brasil; II Universidade de São Paulo Faculdade de Saúde Pública Departamento de Saúde, Ciclos de Vida e Sociedade São PauloSP Brasil Universidade de São Paulo. Faculdade de Saúde Pública. Departamento de Saúde, Ciclos de Vida e Sociedade. São Paulo, SP, Brasil; III Universidade Católica de Santos Departamento de Epidemiologia SantosSP Brasil Universidade Católica de Santos. Programa de Pós-Graduação em Saúde Coletiva. Departamento de Epidemiologia. Santos, SP, Brasil; IV Stockholm University Stress Research Institute Department of Psychology Stockholm Sweden Stockholm University. Stress Research Institute. Department of Psychology. Stockholm, Sweden

**Keywords:** Faculty, Universities, Scientific and Technical Activities, Academic Success, Efficiency, Organizational, Working Conditions, Job Satisfaction, Occupational Health

## Abstract

**OBJECTIVE:**

To assess the association between the perception of pressure to publish academic work with job satisfaction and stress.

**METHODS:**

Cross-sectional study with 64 graduate advisors from a public university in the city of São Paulo. Data collection conducted via an online questionnaire that included: sociodemographic, work and health data; Occupational Stress Indicator Job Satisfaction Scale and Effort-Reward Imbalance (ERI) model. To assess the perception of pressure to publish academic work the advisors answered a numerical scale, assigning a score from 0 to 10 to how pressured they felt to publish their work (being 0 no pressure and 10 high pressure). Later, the generalized linear model was used to test the factors associated to high perception of pressure to publish academic work, adjusted for working time, academic management role and productivity grant.

**RESULTS:**

Advisors who had already worked in a higher education institution, who performed part of the work at home and who reported work stress were more likely to show perception of extreme pressure to publish academic work. This perception was associated with greater effort and over-commitment, as well as a greater imbalance between the effort employed and the reward received at work.

**CONCLUSIONS:**

The findings suggest that the professors’ work organization and mental health are interrelated: the higher the perception of pressure to publish academic work, the greater the stress. However, this result does not seem to be reflected in the job satisfaction (or dissatisfaction). The apparently deliberate extension of working hours hides the precariousness and increased work to which professors have been subjected in recent years by public policies that commercialize education in Brazil.

## INTRODUCTION

Productivity as a central goal of work activity is explicit in means of production of material goods. However, with the hegemony of neoliberal logic, the focus on productivity has been featuring in predominantly intellectual jobs, such as university teaching. A new work organization was established in public universities, affecting teaching practice, their professional and social relations^1^^,^^2^, resulting in precariousness, increased and flexible work, peer competitiveness and pressure for quantitative performance^3^. Working time is reconfigured to meet productivity requirements, leading professors to rush their activities^4^ and extend their time at work^5^.

From the materialization of scientific production in articles and/or books, teaching efficiency and productivity are now indexed and, consequently, subjected to quantitative goals, equivalent to the production sector of material goods. Publications are highlighted in the teaching work, as they guarantee part of the salaries and good evaluations of graduate programs (PPG), besides allowing to maintain positions and ascend in the academic career, as well as obtain research funding^6^^,^^7^.

This context incorporates the concept of academic productivism, defined as a “phenomenon usually derived from official or unofficial regulatory and control processes, supposedly evaluative, characterized by the excessive valuation of the quantity of scientific-academic production, tending to disregard its quality” (SGUISSARDI^8^, 2010, p.01).

Some studies have assessed the effects of bibliographic productivity using interviews and qualitative analyses, pointing to the pressure to publish academic work as an overload factor^9^ and generating suffering and illness^10^, being intrinsically related to the professionals’ physical and mental health^11^. Therefore, it is one of the psychosocial aspects that produce occupational stress^12^. However, until the date of completion of this study, we found no quantitative studies on the topic.

Considering the transformations in the teaching activity, resulting from changes in education policies, and the impacts of this new dynamics of work organization, this study aims to verify the association between the perception of pressure to publish academic work with job satisfaction and stress, under the hypothesis that the higher the perception of pressure to publish reported, the greater the chance of job dissatisfaction and stress.

## METHODS

This is a cross-sectional, non-probabilistic study, conducted from October to December 2018, in which professors from a public university in São Paulo were invited to participate by completing a self-administered questionnaire, initially sent online, given practicality and low cost. According to the inclusion criteria, participants should have been acting as graduate advisors for three years or more. Professors with less than three years of service and, therefore, in the probationary phase, were excluded from the research. Later, to minimize losses, the professors were contacted in person, and the printed questionnaire was made available to the interested parties.

The sociodemographic and work questionnaire was adapted from the Teaching Work Study Protocol^10^ with the following variables surveyed: gender; age; children; residence; previous job; accredited PPG; faculty affiliation, category and work regime; length of service; academic management; productivity grant; activities outside of role assignments; workplace; working hours and lunch break, reading of e-mail and work-related materials; undergraduate and PPG advisees; coordination of extension projects; funded research; issuance of an opinion; participation in examination boards and conventions and link with foreign university. Until the end of the research, we found no equivalent scale in the literature that classified the perception of pressure for academic productivity. Thus, we developed a scale where professors assigned a score from 0 to 10 to their perception of pressure for productivity (exposure) considering 0 as no pressure and 10, a lot of pressure (independent variable). To analyze the associations, we dichotomized the variable by the mean, considering values from 0 to 7.9 as “low pressure” and values from 8 to 10 as “high pressure.”

Satisfaction (outcome) was measured by the translated and validated Portuguese version of the Occupational Stress Indicator Job Satisfaction Scale (OSI)^13^. This scale measures the feeling of satisfaction/dissatisfaction from the subjects’ perception about 22 work psychosocial aspects, using a six-point Likert scale, with final score of 22 to 132 points. For descriptive analysis, the variables were categorized as: dissatisfaction (“very dissatisfied” and “less than satisfied”), intermediate (“somewhat dissatisfied” and “somewhat satisfied”) and satisfaction (“satisfied” and “very satisfied”). As it lacked a cut-off point, the scale was organized in tertile in the association analyses, with the lower third classified as “Dissatisfied” and the upper third “Satisfied.” OSI Cronbach's alpha was 0.94, indicating excellent internal consistency of the scale.

The stress-generating work situations were evaluated by the Effort-Reward Imbalance Questionnaire (ERI), using the translated and validated Portuguese version^14^. Considered a good predictor of physical and psychological health in different occupational groups, this model is pertinent to assess the different stressful factors to which professors are exposed^15^. It is based on the sociological hypothesis of work reciprocity, considering occupational stress as a result of an imbalance between the effort employed and the reward received at work. A third psychometric scale composes the model, called Over-commitment. It is an intrinsic component to assess effort, related to personal motivation so that people respond to job demands with higher motivation and expectations than usual, assuming more responsibilities^16^. Effort and reward scores were obtained by adding the score equivalent to each question, without a pre-established cutoff point. The commitment score was obtained from the sum of the questions, considering values equal to or higher than 18 points as “high over-commitment”^16^. The version used in this research contains 23 questions, excluding the one referring to physical exertion, given the predominantly intellectual characteristic of the teaching activity^17^.

The imbalance between effort and reward (outcome) was assessed by the algorithm e/(rxc), in which “e” represents the effort score, divided by the reward score (“r”) multiplied by the coefficient “c” (number of effort questions divided by the reward questions). Values close to 0 indicate favorable condition (low effort/high reward) and above 1, unfavorable condition (great effort/low reward)^16^. Cronbach's alpha of the ERI model coefficient was 0.90 and of the effort, reward and commitment scales was 0.71, 0.90 and 0.85, respectively, all presenting an excellent internal consistency.

Data descriptive analysis by means, standard deviation, minimum and maximum values when parametric, and median and interquartile range – IQR (P25-P75) when non-parametric was used to characterize the sample; categorical variables were described in absolute and relative frequencies. The normality of the variables was tested using the Shapiro-Wilk test and, in the absence of normality, by the Spearman's correlation coefficient. Pearson's chi-square hypothesis tests and Fischer's exact hypothesis were used to compare proportions.

To answer the research question, the means of the exposure variable “perception of pressure to publish academic work” were compared with the outcome variables “job satisfaction” and “ERI” using generalized linear models (GLM). The adjustment variables were: length of service in the institution (in years), since length of teaching can interfere in the perception of stress; academic management (performs or not), as they are activities performed by 87.5% of professors and can encumber the work; and productivity grant (owns or not), considering that monetary stimulus can lead workers to extrapolate their individual tolerance limits. The study was approved by the ethics committee and conducted after the participants’ informed consent (CAAE: 88460618.7.0000.5421).

## RESULTS

Of the 178 invited professors, 112 refused to participate and two were excluded for being in the probationary stage, totaling a sample of 64 participants (46 online and 18 printed answers). The unavailable data from the professors who refused to participate in the research hindered analyzing the losses in detail. Most professors were women (62.5%), with a median age of 58 years (IQR = 47.9–63.7 years). There was no significance in the statistical tests using the variable gender with the perception of pressure to publish academic work. The professors were distributed in 14 PPG, 50% of which accredited in a public health program. The median length of service was 15.4 years (IQR = 8.2–28 years), with 98% being full professor. Around 80% reported working on weekends (median of 4.2 hours; IQR = 3.4–8 hours) and more than 78% performed part of the academic work at home (median of 10 hours; IQR = 3.1–16 hours), practice justified by high job demand (59.4%), short deadlines (50%) and fewer home interruptions (40.6%). Regarding working time distribution, 65.6% reported dedicating 30 minutes to one hour to their lunch break, one to three hours to reading e-mail (58.7%) and more than two hours per week to reading work related materials (46%). The median of classes taught in the first semester of 2018 was eight classes (IQR = 5.5–12 classes), as well as in the second semester (IQR = 5–12 classes). More than 87% reported performing roles related to academic management; 51.6% coordinated extension projects; 78.1% had publicly funded research and 50% lacked a productivity grant from the National Council for Scientific and Technological Development (CNPq). All participants were advisors, with a median of two undergraduate students (IQR = 1–3 students) and 5.6 graduate students (SD = 2.9 students) under their supervision.

Around 50% of professors reported performing activities outside their duties, justified by the insufficient number of employees. More than half of the professors had previously worked in higher education institutions (HEI), 65% of them in private institutions. In the last two years, 44% of the professors attended at least one convention presenting papers, and 57.8% participated in two to five examination boards. Almost 94% of the participants issued an opinion to journals, events and/or research projects. In the last two years, the median of published articles was 6 (IQR = 3–11 articles), with a maximum of 42; 0 book chapters (IQR = 0–2.8 chapters), with a maximum of 16 publications and 0 books (IQR = 0–0 books), with a maximum of two publications. More than half of the professors gave a score of 9 and 10 to the perception of pressure to publish academic work.

**Figure 1:**
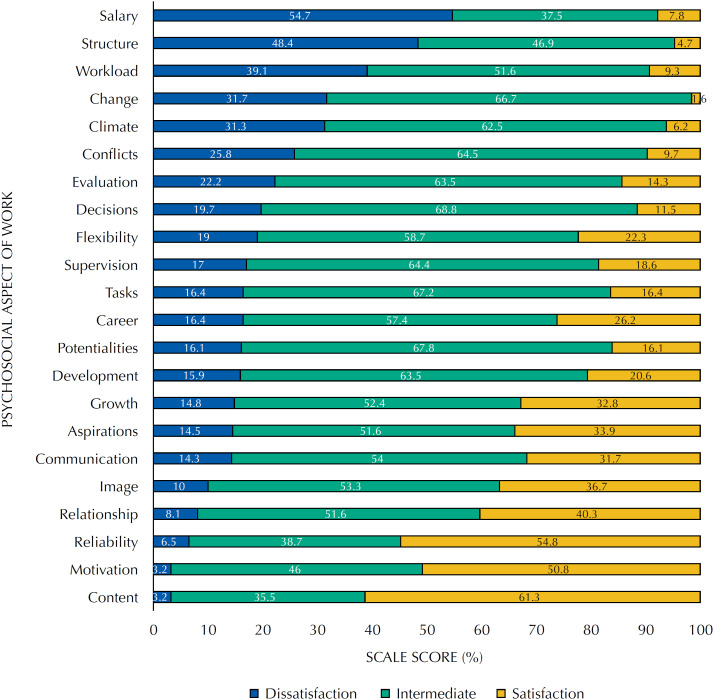
Distribution of the percentage of professors according to the degree of satisfaction and aspects of the work.

Regarding job satisfaction, the average was 78.9 points (SD = 16.3), with emphasis on “salary in relation to experience and responsibility,” “organizational structure” and “workload” as the main aspects that generate dissatisfaction (Figure 1).

**Figure 2:**
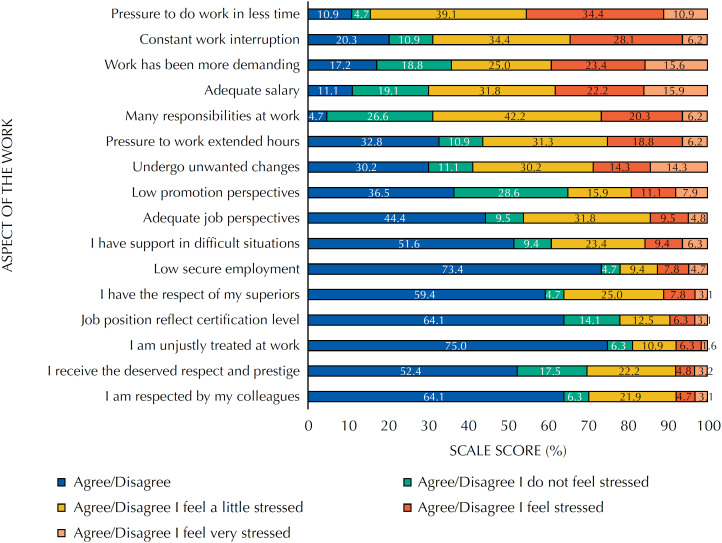
Distribution of the percentage of professors according to the effort-reward imbalance model (ERI) and aspect of the work.

According to the ERI model, 85% of the professors were in an imbalance, suggesting work stress, and 23.4% showed high over-commitment. Figure 2 shows the distribution of the percentages of answers in each question of the ERI questionnaire.

There was no statistically significant association between job satisfaction and perception of pressure to publish academic work. Professors who previously worked in HEI (76.5%), performed part of the work at home (72%) and reported occupational stress (65.1%) were more likely to feel this pressure (Table).

**Table:** Characterization of the perception of pressure to publish academic work according to sociodemographic and work-related variables by Pearson's chi-square test, São Paulo, 2018.

Variable		Total n	Low pressure n (%)	High pressure n (%)	χ^2^ p-value
Gender		64			0.13
	Male		11(45.8)	13 (54.2)	
	Female		11(27.5)	29 (72.5)	
Length of service at USP		64			0.07
	Below average		10(25.6)	29 (74.4)	
	Above average		12(48)	13 (52)	
Academic management		64			0.73^1^
	Do not perform		3 (27.3)	8 (72.7)	
	Performs		19 (35.8)	34 (64.2)	
Certification		64			0.77^a^
	Doctor		3 (27.3)	8 (72.7)	
	PhD		2 (25)	6 (75)	
	Associate professor		17 (37.8)	28 (62.2)	
Previous HEI		64			0.05^c^
	No		14 (46.7)	16 (53.3)	
	Yes		8 (23.5)	26 (76.5)	
Productivity grant		64			0.75^a^
	No		10 (31.2)	22 (68.8)	
	Yes		10 (45.5)	15 (35.7)	
	I've had it, I don't have any more		2 (9)	5 (11.9)	
Productivity grant b		64			0.45
	No		12 (54.5)	27 (64.3)	
	Yes		10 (40)	15 (60)	
Workplace		64			0.04^c^
	University		8 (57.1)	6 (42.9)	
	University/home		14 (28)	36 (72)	
Workload		62			0.77
	Below average		12 (32.4)	25 (67.6)	
	Above average		9 (36)	16 (64)	
Works in the weekend/holiday		64			0.12
	No		6 (54.6)	5 (45.4)	
	Yes		16 (30.2)	37 (69.8)	
Funded research		64			0.22
	Yes		6 (25)	18 (75)	
	No		16 (40)	24 (60)	
Emotional disorders		64			0.25
	Do not have		14 (43.7)	18 (56.3)	
	Has – self-diagnosis		3 (20)	12 (80)	
	Has – diagnostic		5 (29.4)	12 (70.6)	
Job satisfaction/tercile		55			0.18^a^
	Dissatisfaction		4 (22.2)	14 (77.8)	
	Intermediate		4 (22.2)	14 (77.8)	
	Satisfaction		9 (47.4)	10 (52.6)	
ERI Coefficient		62			0.05^a^.^c^
	Balance		6 (66.7)	3 (33.3)	
	Imbalance		15 (34.9)	28 (65.1)	

a Fisher's Exact Test

b Dichotomized variable of having a productivity grant and adding those who do not have with those who have already had it.

c p < 0.05

**Figure 3:**
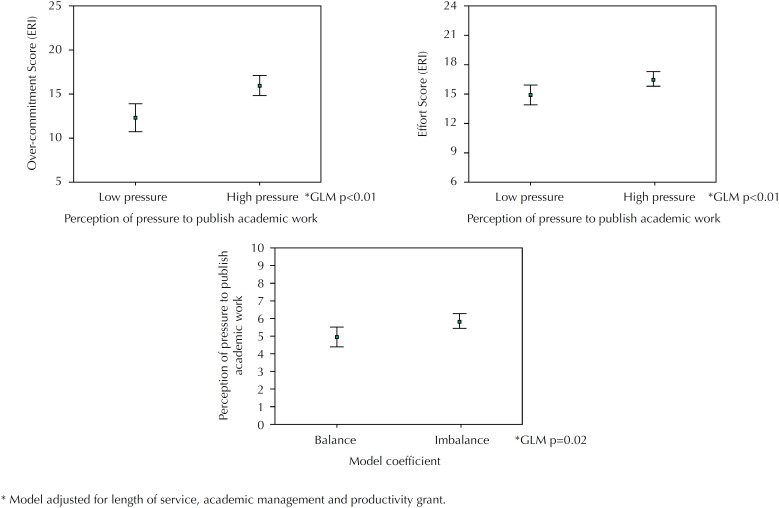
Comparison of means and confidence interval (95%) of the effort, over-commitment and coefficient (stress) scores of the effort-reward imbalance model (ERI), with the perception of pressure to publish academic work.

Using GLM, we found an association between the perception of pressure to publish academic work and the means of effort, over-commitment and effort-reward coefficient, adjusted for length of service in the institution, academic management role and productivity grant (Figure 3).

## DISCUSSION

This study verified the association between perception of pressure to publish academic work and stress, effort and over-commitment (ERI model), regardless of work-related variables. We found no association between the exposure variable and job satisfaction. Among university professors, research has been pointed as triggering occupational stress^18^^,^^19^ and the task where the increased work is most felt^20^ – consequences of the productivity culture that prevails in universities. Such association can be understood within the ERI model theoretical framework, in which the perception of a great degree of effort in meeting the demands (overload) is not accompanied by the perception of equivalent reward. This unfavorable perception may be associated with the professors’ salaries, referred to by the research participants as the psychosocial aspect that causes more dissatisfaction (54.7%) and stress (15.9%).

The perception of pressure to publish academic work appeared associated with the ERI effort variable, a component that proposes to measure job demand. Studies have discussed the effects of bibliographic productivity on the lives of university professors, suggesting it as a precursor of suffering and illness^10^ and that reduces the quality of work^4^. In our study, half of the professors were in a PPG with grade 6 or 7 on the 2017 evaluation of the *Coordenação de Aperfeiçoamento de Pessoal de Nível Superior* (CAPES); the others, in programs with a minimum score of 4. It should be emphasized that 50% of this assessment is based on the teaching staff and its intellectual production^21^. The average number of publications by the research participants was 5.1 in 2017, almost 50% above the general average of the institution's professors in the same year^22^. Information from the institution's database shows an 18.2% increase in the number of articles indexed in the Web of Science, against a 6.3% reduction in the number of professors between 2013 and 2017, suggesting an increased work regarding intellectual production. Studies question the real accuracy of an academic performance assessment measured by bibliographic indicators, as well as the consequences for the authors’ health and the quality of the works written^1^^,^^23^^–^^25^^,^^12^.

Scientific publications materialize part of the teaching practice. Given the predominantly immaterial characteristic of this type of job, a large portion of its tasks require a considerable time investment and lacks the prominence attributed to publications (such as reading e-mails, participating in thesis presentations, among others). There is also an invisible part of the work, evident by how most professors have to perform tasks outside their role, that is, non-prescribed activities whose solution requires additional time.

Thus, bibliographic productions are not only one of the professors’ many demands, but occupy a prominent role in academic work. Allied to the numerous demands of the job, professors can perceive them as psychic overload, experiencing a feeling of greater effort in fulfilling this activity.

**Figure 4:**
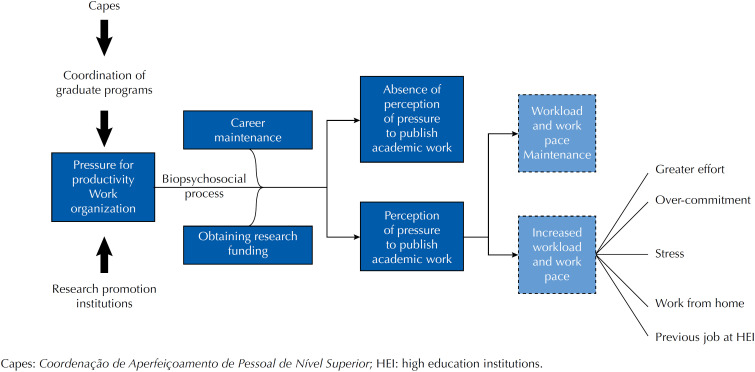
Hypothetical trigger model of the perception of pressure to publish academic work and its associations.

Over-commitment also appeared associated with the perception of pressure to publish academic work, regardless of work-related variables. This association can be explained by a subjective path of effort, different from the previous ones, which were mediated by the work organization. Participants with this characteristic tend to underestimate job demands and overestimate their coping strategies, resulting in over-commitment combined with a desire for recognition and esteem^16^. To understand the trigger mechanism of the association between these variables, we proposed the following hypothetical model (Figure 4).

In the mechanism, the pressure for bibliographic productivity comes from the CAPES and PPG, which adopt this item to evaluate individual teaching performance (which may generate promotion or loss of accreditation) and as part of a larger evaluation that classifies the PPGs. Publications also condition obtaining financial resources for research, which generates new publications and contributes to maintaining the career, in a cyclical process. The perception of pressure to publish academic work emerges from these conditions, as the result of a biopsychosocial process. The pressure for productivity as a perception is not universal, given its subjective characteristic, and individual differences should be considered in the strategies to face the adversities imposed by the organization. This process can trigger increased workload and work pace, associated with the perception of greater work effort and commitment, as well as occupational stress, the habit of taking tasks home and previous job in HEI.

Needing to perform part of the work at home was a factor associated with the perception of pressure to publish academic work, which may be justified by the workload combined with the short deadlines. This conflation results in increased work pace (self-acceleration), which forces professors to reorganize their time, subtracting hours of rest. This intensification, although naturalized, has been associated with physical and social consequences predictors of chronic stress^26^. In line with these findings are the studies by Mendonça-Lima and Lima Filho^27^, which point to work overload as responsible for teaching activity on weekends and vacation periods.

Previous work in HEI was also associated with the perception of pressure to publish academic work in the present study. An explanatory hypothesis for this finding concerns the differences in work organization between private and public HEI (78.5% of the professors had worked in private HEI). In public HEIs, bibliographic production is higher than in private HEIs^28^. In changing institutions, the productivity culture may result in work overload.

We found no statistically significant association between job dissatisfaction and perception of pressure to publish academic work. Perhaps the gratification in the teaching career overcomes the negative perception of set goals, due to the impersonal and not immediately “interested” nature of the work, enabling self-realization^29^. We must also consider the low sample number.

It is worth noting that the cross-sectional design of the study precludes establishing a causal relationship between work psychosocial factors and the perception of pressure to publish academic work. The non-probabilistic nature of the sample may have created a participation bias, in which teachers with higher perceptions of pressure for job demands had a greater interest in participating in the research. Finally, the low turnout (approximately 37% of the population) can be explained by the period we conducted the survey (October to December 2018). According to the professors themselves, the end of the year is the time when the workload increases, and deadlines expire.

Our findings suggest that the professors’ work organization and mental health are interrelated, drawing attention to academic management, which has been insufficiently considered in studies on mental health at work. It is important to highlight that, although all analyses considered stress as a dependent variable, it is impossible to categorically state the direction of the association.

In summary, the higher the perception of pressure to publish academic work, the greater the stress at work. However, this result does not seem reflect in job satisfaction or dissatisfaction. Extending working hours is apparently a deliberate choice by the professor, but it hides the precariousness and increased work to which professors, both from universities and basic education, have been subjected in recent years by public policies that commercialize education in Brazil.
