# Supplementary material for: Academic productivism: when job demand exceeds working time
Source: Rev Saude Publica. 2020 Nov 17;54:117. doi: 10.11606/s1518-8787.2020054002288 (PMC7671584; doi:10.11606/s1518-8787.2020054002288)
Supplement: Translation

# Translation: Produtivismo acadêmico: quando a demanda supera o tempo de trabalho

**Authors:** Talita da Silveira Campos Teixeira, Elaine Cristina Marqueze, Claudia Roberta de Castro Moreno

**Affiliations:** I São PauloSP Brasil Universidade de São Paulo. Faculdade de Saúde Pública. Programa de Pós-Graduação em Saúde Pública. São Paulo, SP, Brasil; II São PauloSP Brasil Universidade de São Paulo. Faculdade de Saúde Pública. Departamento de Saúde, Ciclos de Vida e Sociedade. São Paulo, SP, Brasil; III SantosSP Brasil Universidade Católica de Santos. Programa de Pós-Graduação em Saúde Coletiva. Departamento de Epidemiologia. Santos, SP, Brasil; IV Stockholm Sweden Stockholm University. Stress Research Institute. Department of Psychology. Stockholm, Sweden

**Keywords:** Docentes, Universidades, Atividades Científicas e Tecnológicas, Sucesso Acadêmico, Eficiência Organizacional, Condições de Trabalho, Satisfação no Emprego, Saúde do Trabalhador

## Abstract

**OBJETIVOS:**

Avaliar a associação entre a percepção da pressão por publicações com a satisfação e o estresse no trabalho.

**MÉTODOS:**

Estudo transversal com 64 orientadores de pós-graduação de uma universidade pública da cidade de São Paulo. A coleta de dados ocorreu por meio de um questionário on-line que incluiu: dados sociodemográficos, laborais e de saúde; Escala de Satisfação no Trabalho do Occupational Stress Indicator e modelo Desequilíbrio Esforço-Recompensa (DER). Para avaliar a percepção da pressão por publicação foi criada uma escala numérica na qual o orientador deveria atribuir uma nota de 0 a 10 para o quanto se sentia pressionado a publicar seus trabalhos (sendo 0 nenhuma pressão e 10 muita pressão). Posteriormente, foi utilizado o modelo linear generalizado para testar os fatores associados à alta percepção de pressão para publicação, ajustado pelo tempo de trabalho, função de gestão acadêmica e bolsa produtividade.

**RESULTADOS:**

Maiores proporções da percepção de elevada pressão para publicação foram encontradas entre orientadores que já haviam trabalhado em instituição de ensino superior, que realizavam parte do trabalho em casa e que apresentavam estresse laboral. Associou-se essa percepção a um maior esforço e comprometimento excessivo no trabalho, bem como a um maior desequilíbrio entre o esforço empregado e a recompensa recebida no trabalho.

**CONCLUSÕES:**

Os achados desta pesquisa sugerem que a organização do trabalho e a saúde mental dos trabalhadores estejam inter-relacionados: quanto maior a percepção de pressão por publicação maior o estresse. No entanto, esse resultado parece não se refletir na satisfação (ou insatisfação) do trabalho. O prolongamento aparentemente deliberado das horas de trabalho oculta a precarização e intensificação do trabalho a que os professores têm sido submetidos nos últimos anos pelas políticas públicas que mercantilizam a educação no Brasil.

## INTRODUÇÃO

A produtividade como meta central da atividade de trabalho é explícita em meios de produção de bens materiais. Entretanto, com a hegemonia da lógica neoliberal, a centralidade na produtividade tem aportado em trabalhos predominantemente intelectuais, como é o caso da docência universitária. Uma nova organização do trabalho estabelece-se nas universidades públicas, afetando a prática docente, suas relações profissionais e sociais^1^^,^^2^, gerando consequências como precarização, intensificação e flexibilização do trabalho, competitividade entre pares e pressão pelo desempenho quantitativo^3^. O tempo de trabalho reconfigura-se para atender as exigências produtivistas, levando o docente a acelerar suas atividades^4^ e prolongar o tempo no trabalho^5^.

A partir da materialização da produção científica em artigos e/ou livros, a eficiência e a produtividade docente passam a ser objetivadas em índices e, consequentemente, subordinadas a metas quantitativas, equivalendo-se aos setores de produção de bens materiais. As publicações assumem destaque no trabalho docente, à medida que garantem parte das remunerações e boas avaliações dos programas de pós-graduação (PPG), além de permitirem conservar cargos e ascender na carreira, bem como obter financiamento de pesquisa^6^^,^^7^.

Nesse contexto, incorpora-se a concepção do produtivismo acadêmico, definido como um “fenômeno em geral derivado dos processos oficiais ou não de regulação e controle, supostamente de avaliação, que se caracteriza pela excessiva valorização da quantidade da produção científico-acadêmica, tendendo a desconsiderar a sua qualidade” (SGUISSARDI^8^, 2010, p.01).

Alguns estudos têm avaliado os efeitos da produtividade bibliográfica por meio de entrevistas e análises qualitativas, apontando a pressão por publicação como fator de sobrecarga^9^ e geradora de sofrimento e adoecimento^10^, estando intrinsecamente relacionada à saúde física e mental dos profissionais^11^. Portanto, trata-se de um dos aspectos psicossociais geradores de estresse ocupacional^12^. No entanto, até a data de conclusão deste estudo não encontramos pesquisas quantitativas relacionadas ao tema.

Considerando as transformações na atividade docente, advindas de mudanças nas políticas de educação, e os impactos dessa nova dinâmica da organização do trabalho, este estudo se propõe a verificar a existência de associação entre a percepção da exigência por publicações com a satisfação e o estresse no trabalho, sob a hipótese de que quanto maior a percepção de pressão por publicação referida pelo docente, maior a chance de insatisfação e estresse no trabalho.

## MÉTODOS

Trata-se de estudo transversal, não probabilístico, realizado de outubro a dezembro de 2018, em que docentes de uma universidade pública de São Paulo foram convidados a participar por meio do preenchimento de um questionário autoaplicável, enviado inicialmente on-line, dada a praticidade e o baixo custo. Segundo critério de inclusão, os participantes deveriam estar há três anos ou mais como orientadores de pós-graduação. Docentes com menos de três anos de trabalho e, portanto, em estágio probatório constitucional, foram excluídos da pesquisa. Posteriormente, a fim de minimizar as perdas, os docentes foram contatados pessoalmente, e o questionário impresso foi disponibilizado aos interessados.

O questionário sociodemográfico e laboral foi adaptado do Protocolo de Estudos sobre o Trabalho Docente^10^ com as seguintes variáveis pesquisadas: sexo; idade; filhos; residência; trabalho anterior; PPG credenciado; modalidade de vínculo, categoria e regime de trabalho; tempo de trabalho; gestão acadêmica; bolsa produtividade; atividades fora das atribuições de função; local da jornada; horas de trabalho e distribuição do tempo para almoço, leitura de e-mail e de materiais relacionados ao trabalho; orientandos na graduação e em PPG; coordenação de projetos de extensão; pesquisas financiadas; emissão de parecer; participação em bancas e congressos e vínculo com universidade estrangeira. Até o encerramento da pesquisa, não foi encontrada na literatura uma escala equivalente que classificasse a percepção da cobrança por produtividade acadêmica. Assim, criou-se para este estudo uma escala em que o docente deveria atribuir nota de 0 a 10 para sua percepção de cobrança por produtividade (exposição) considerando 0 como nenhuma pressão e 10, muita pressão (variável independente). Para análise das associações, a variável foi dicotomizada pela média, considerando valores de 0 a 7,9 como “baixa pressão” e valores de 8 a 10 como “alta pressão”.

A satisfação (desfecho) foi mensurada por meio da versão traduzida e validada para língua portuguesa da Escala de Satisfação no Trabalho do Occupational Stress Indicator (OSI)^13^. Essa escala mensura o sentimento de satisfação/insatisfação a partir da percepção dos sujeitos sobre 22 aspectos psicossociais do trabalho, utilizando uma escala de Likert de seis pontos, com escore final de 22 a 132 pontos. Para análise descritiva, as variáveis foram categorizadas em: insatisfação (“enorme insatisfação” e “muita insatisfação”), intermediário (“alguma insatisfação” e “alguma satisfação”) e satisfação (“muita satisfação” e “enorme satisfação”). Por não possuir ponto de corte, a escala foi organizada em tercil nas análises de associação, sendo o terço inferior classificado como “Insatisfeitos” e o superior, “Satisfeitos”. O alfa de Cronbach do OSI foi de 0,94, indicando ótima consistência interna da escala.

As situações de trabalho geradoras deestresse foram avaliadas pelo modelo Desequilíbrio-Esforço-Recompensa (DER) (Effort-Reward Imbalance Questionnaire), utilizando a versão traduzida e validada para língua portuguesa^14^. Esse modelo é considerado um bom preditor da saúde física e psicológica em diferentes grupos ocupacionais, sendo pertinente para estudar os diversos fatores estressantes aos quais os docentes estão expostos^15^. O modelo se baseia na hipótese sociológica da reciprocidade no trabalho, considerando o estresse ocupacional como resultado de um desequilíbrio entre o esforço empregado e a recompensa recebida no trabalho. Uma terceira escala psicométrica compõe o modelo, chamada Comprometimento Excessivo. Trata-se de um componente intrínseco à avaliação do esforço, relacionada à motivação individual para que as pessoas respondam às demandas de trabalho com maiores motivações e expectativas do que de costume, assumindo mais responsabilidades^16^. Os escores de esforço e recompensa foram obtidos pela soma da pontuação equivalente a cada questão, sem um ponto de corte pré-estabelecido. Obteve-se o escore do comprometimento a partir da somatória das questões, considerando valores iguais ou maiores que 18 pontos como “alto comprometimento excessivo”^16^. A versão utilizada nesta pesquisa contém 23 questões, sendo excluída a pergunta referente ao esforço físico, dada a característica predominantemente intelectual da atividade docente^17^.

O desequilíbrio entre esforço e recompensa (desfecho) foi avaliado a partir do algoritmo e/(rxc), em que “e” representa o escore do esforço, divido pela multiplicação entre o escore da recompensa (“r”) e o coeficiente “c” (número de questões do esforço dividido pelo de recompensa). Valores próximos a 0 indicam condição favorável (baixo esforço/alta recompensa) e acima de 1, condição desfavorável (grande esforço/baixa recompensa)^16^. O alfa de Cronbach do coeficiente do modelo DER foi de 0,90 e das escalas de esforço, recompensa e comprometimento foi de 0,71, 0,90 e 0,85, respectivamente, em que todos apresentaram uma ótima consistência interna.

Para a caracterização da amostra utilizou-se a análise descritiva dos dados por meio de médias, desvio-padrão, valores mínimos e máximos quando paramétricas, e mediana e amplitude interquartílica – AIQ (P25–P75) quando não paramétricas; as variáveis categóricas foram descritas em frequências absolutas e relativas. Testou-se a normalidade das variáveis por meio do teste de *Shapiro-Wilk* e, a partir desta informação, do Coeficiente de Correlação de *Spearman*, na ausência de normalidade. Os testes de hipótese de qui-quadrado de Pearson e Exato de Fischer foram utilizados para comparar proporções.

Para responder à pergunta de pesquisa, compararam-se as médias da variável de exposição “percepção da pressão por publicação” com as variáveis desfecho “satisfação no trabalho” e “DER” por meio de modelos lineares generalizados (GLM). As variáveis de ajuste foram: tempo de trabalho na instituição (em anos), já que o tempo de docência pode interferir na percepção do estresse; gestão acadêmica (realiza ou não), pois são atividades realizadas por 87,5% dos docentes que podem onerar o trabalho; e bolsa produtividade (possui ou não), considerando que o estímulo pecuniário pode levar o trabalhador a extrapolar limites de tolerância individual. O estudo foi aprovado pelo comitê de ética e realizado após o consentimento livre e esclarecido dos participantes (CAAE: 88460618.7.0000.5421).

## RESULTADOS

Dos 178 docentes convidados, 112 se recusaram a participar e dois foram excluídos do estudo, pois estavam em estágio probatório constitucional, totalizando uma amostra de 64 docentes (sendo 46 respostas on-line e 18 impressas). A indisponibilidade de dados dos docentes que se recusaram a participar da pesquisa inviabilizou uma análise detalhada das perdas. A maioria dos professores era mulher (62,5%), sendo a mediana de idade de 58 anos (AIQ = 47,9–63,7 anos). Não houve significância nos testes estatísticos utilizando a variável sexo com a percepção da pressão por publicação. Os docentes estavam distribuídos em 14 PPG, sendo 50% credenciados em um programa de saúde pública. A mediana do tempo de trabalho foi de 15,4 anos (AIQ = 8,2–28 anos), com 98% em regime de dedicação exclusiva. Aproximadamente 80% relataram trabalhar nos fins de semana (mediana de 4,2 horas; AIQ = 3,4–8 horas) e mais de 78% realizavam parte do trabalho acadêmico em casa (mediana de 10 horas; AIQ = 3,1–16 horas), prática justificada por elevada demanda de trabalho (59,4%), prazos exíguos (50%) e menos interrupções no domicílio (40,6%). Quanto à distribuição do tempo de trabalho, 65,6% relataram dedicar de 30 minutos a uma hora para seu almoço, de uma a três horas para leitura de e-mail (58,7%) e mais de duas horas por semana para leitura de materiais relacionados ao seu trabalho (46%). A mediana de aulas dadas no primeiro semestre de 2018 foi de oito aulas (AIQ = 5,5–12 aulas), assim como no segundo semestre (AIQ = 5–12 aulas). Mais de 87% relataram exercer funções relacionadas à gestão acadêmica; 51,6% coordenavam projetos de extensão; 78,1% tinham pesquisas com financiamento público e 50% não possuíam bolsa produtividade do Conselho Nacional de Desenvolvimento Científico e Tecnológico (CNPq). Todos os participantes eram orientadores, com mediana de dois graduandos (AIQ = 1–3 alunos) e 5,6 pós-graduandos (DP = 2,9 alunos) sob sua orientação.

Aproximadamente 50% dos docentes relataram realizar atividades fora de suas atribuições, justificadas pelo número insuficiente de funcionários. Mais da metade dos professores já havia trabalhado anteriormente em instituições de ensino superior (IES), 65% deles em instituições particulares. Nos últimos dois anos, 44% dos docentes estiveram em ao menos um congresso apresentando trabalhos, e 57,8% estiveram entre duas a cinco bancas. Quase 94% dos participantes emitiam parecer para periódicos, eventos e/ou projetos de pesquisa. Nos últimos dois anos, a mediana de artigos publicados foi de 6 (AIQ = 3–11 artigos), com máximo de 42; 0 capítulos de livros (AIQ = 0–2,8 capítulos), com máximo de 16 publicações e 0 livros (AIQ = 0–0 livros), com máximo de duas publicações. Mais da metade dos docentes atribuiu nota 9 e 10 para a percepção de pressão por publicação.

Quanto à satisfação no trabalho, a média foi de 78,9 pontos (DP = 16,3), com destaque para “salário em relação à experiência e à responsabilidade”, “estrutura organizacional” e “volume de trabalho” como principais aspectos geradores de insatisfação (Figura 1).

**Figura 1:** Distribuição da porcentagem de docentes segundo grau de satisfação e aspectos do trabalho.

De acordo com o modelo DER, 85% dos docentes estavam em situação de desequilíbrio, sugerindo estresse laboral, e 23,4% classificados com alto comprometimento excessivo. A Figura 2 apresenta a distribuição dos percentuais de respostas em cada pergunta do questionário do DER.

**Figura 2:** Distribuição da porcentagem de docentes segundo o modelo de desequilíbrio entre esforço e recompensa (DER) e aspecto do trabalho.

Não houve associação estatisticamente significante entre a satisfação no trabalho e a percepção da pressão por publicação. Maiores proporções dessa percepção (Tabela) foram encontradas entre professores com trabalho anterior em IES (76,5%), que realizavam parte do trabalho em casa (72%) e em estresse ocupacional (65,1%).

**Tabela:** Caracterização da percepção da pressão por publicação segundo variáveis sociodemográficas e laborais pelo teste de qui-quadrado de Pearson, São Paulo, 2018.

Variável		Total n	Baixa pressão n (%)	Alta pressão n (%)	χ^2^ valor de p
Sexo		64			0,13
	Masculino		11(45,8)	13 (54,2)	
	Femininos		11(27,5)	29 (72,5)	
Tempo trabalho na USP		64			0,07
	Abaixo da média		10(25,6)	29 (74,4)	
	Acima da média		12(48)	13 (52)	
Gestão acadêmica		64			0,73^a^
	Não exerce		3 (27,3)	8 (72,7)	
	Exerce		19 (35,8)	34 (64,2)	
Titulação		64			0,77^a^
	Doutor(a)		3 (27,3)	8 (72,7)	
	Pós-doutor(a)		2 (25)	6 (75)	
	Livre-docente		17 (37,8)	28 (62,2)	
IES anterior		64			0,05^c^
	Não		14 (46,7)	16 (53,3)	
	Sim		8 (23,5)	26 (76,5)	
Bolsa produtividade		64			0,75^a^
	Não		10 (31,2)	22 (68,8)	
	Sim		10 (45,5)	15 (35,7)	
	Já tive, não tenho mais		2 (9)	5 (11,9)	
Bolsa produtividade^b^		64			0,45
	Não		12 (54,5)	27 (64,3)	
	Sim		10 (40)	15 (60)	
Local jornada		64			0,04^c^
	Universidade		8 (57,1)	6 (42,9)	
	Universidade/casa		14 (28)	36 (72)	
Carga horária		62			0,77
	Abaixo da média		12 (32,4)	25 (67,6)	
	Acima da média		9 (36)	16 (64)	
Trabalho final semana/feriado		64			0,12
	Não		6 (54,6)	5 (45,4)	
	Sim		16 (30,2)	37 (69,8)	
Pesquisa financiada		64			0,22
	Sim		6 (25)	18 (75)	
	Não		16 (40)	24 (60)	
Distúrbio emocional		64			0,25
	Não possui		14 (43,7)	18 (56,3)	
	Possui – opinião		3 (20)	12 (80)	
	Possui – diagnóstico		5 (29,4)	12 (70,6)	
Satisfação no trabalho/tercil		55			0,18^a^
	Insatisfação		4 (22,2)	14 (77,8)	
	Intermediário		4 (22,2)	14 (77,8)	
	Satisfação		9 (47,4)	10 (52,6)	
Coeficiente DER		62			0,05^a^,^c^
	Equilíbrio		6 (66,7)	3 (33,3)	
	Desequilíbrio		15 (34,9)	28 (65,1)	

a Exato de Fischer

b Variável dicotomizada em possuir bolsa produtividade e somando os que não possuem com os que já tiveram.

c p < 0,05

Utilizando GLM, constatou-se associação entre a percepção da pressão por publicação e as médias de esforço, comprometimento excessivo e coeficiente esforço-recompensa, ajustados por tempo de trabalho na instituição, função de gestão acadêmica e bolsa produtividade (Figura 3).

**Figura 3:** Comparação das médias e intervalo de confiança (95%) do escore do esforço, do comprometimento excessivo e do coeficiente (estresse) do modelo de desequilíbrio esforço-recompensa (DER), com a percepção da pressão por publicação.

## DISCUSSÃO

Verificou-se neste estudo a associação entre percepção da pressão por publicação e o estresse, esforço e comprometimento excessivo (modelo DER), independente de variáveis laborais. Não foi encontrada associação entre a variável de exposição e a satisfação no trabalho. Entre docentes universitários, a pesquisa tem sido apontada como desencadeadora de estresse ocupacional^18^^,^^19^ e como a tarefa em que a intensificação do trabalho é mais sentida^20^, consequências da cultura produtivista que prevalece nas universidades. Tal associação pode ser compreendida sob a ótica do referencial teórico do modelo DER, em que a percepção de alto grau de esforço no cumprimento das demandas (sobrecarga) não é acompanhada pela percepção de recompensa equivalente. Essa percepção desfavorável pode estar associada ao salário dos professores, referido pelos participantes desta pesquisa como aspecto psicossocial que causa mais insatisfação (54,7%) e estresse (15,9%).

A percepção da pressão por publicação apareceu associada à variável esforço do modelo DER, componente que se propõe a mensurar as demandas de trabalho. Estudos têm discutido os efeitos da produtividade bibliográfica na vida de docentes universitários, sugerindo-a como precursora de sofrimento e adoecimento^10^ e redutora da qualidade do trabalho^4^. Neste estudo, metade dos docentes estavam em PPG com nota 6 ou 7 pela avaliação de 2017 da Coordenação de Aperfeiçoamento de Pessoal de Nível Superior (Capes); os demais, em programas com nota mínima 4. Ressalte-se que 50% da construção desse conceito pesa sobre o corpo docente e sua produção intelectual^21^. A média de publicações dos participantes desta pesquisa foi de 5,1 em 2017, quase 50% acima da média geral dos docentes da instituição no mesmo ano^22^. Informações do banco de dados da instituição revelam aumento de 18,2% no número de artigos indexados no Web of Science, contra uma redução de 6,3% no número de docentes entre 2013 e 2017, sugerindo intensificação do trabalho no tocante à produção intelectual. Estudos questionam a real acurácia da avaliação do desempenho acadêmico medido por indicadores bibliográficos, bem como as consequências para saúde dos autores e para a qualidade dos trabalhos gerados^1^^,^^23^^–^^25^^,^^12^.

As publicações científicas materializam uma parte da prática docente. Dada a característica predominantemente imaterial desse tipo de trabalho, grande parcela das tarefas demanda considerado investimento de tempo e não recebe o destaque atribuído às publicações (como leitura de e-mail, participação em defesas, entre outras). Existe ainda uma parte invisível do trabalho, evidenciada pela necessidade da maioria dos docentes de realizar tarefas fora de sua função, ou seja, atividades não prescritas cuja solução exige tempo suplementar.

Assim, as produções bibliográficas não são apenas mais uma das muitas demandas dos docentes, mas ocupam papel de destaque no trabalho acadêmico. Aliadas às inúmeras exigências da função, podem ser percebidas como sobrecarga psíquica entre professores, que vivenciam uma sensação de maior esforço no cumprimento desta atividade.

O comprometimento excessivo também apareceu associado à percepção da pressão por publicação, independente de variáveis laborais. Essa associação pode ser explicada por uma via subjetiva de esforço, diferente das anteriores, que eram mediadas pela organização do trabalho. Participantes com essa característica tendem a subestimar as demandas de trabalho e superestimar suas estratégias de enfrentamento, refletindo em esforço excessivo combinado com desejo de reconhecimento e estima^16^. Para compreender o desencadeamento da associação entre as variáveis deste estudo, propõe-se o seguinte modelo hipotético (Figura 4).

**Figura 4:** Modelo hipotético de desencadeamento da percepção da pressão por publicação e suas associações.

No mecanismo, a exigência por produtividade bibliográfica advém da Capes e dos PPG, os quais adotam esse item para avaliar o desempenho docente individual (podendo gerar promoções ou descredenciamento da pós-graduação) e como parte de uma avaliação maior que classifica os PPG. As publicações também condicionam a obtenção de recursos financeiros para pesquisas, o que gera novas publicações e contribui para manter a carreira, num processo cíclico. A percepção da pressão por publicação emerge desses condicionantes, como fruto de um processo biopsicossocial. A cobrança por produtividade enquanto percepção não se aplica a todos, dada sua característica subjetiva, devendo-se considerar as diferenças individuais na estratégia de enfrentamento das adversidades impostas pela organização. Esse processo pode desencadear aumento da carga e do ritmo de trabalho, associado à percepção de maior esforço e comprometimento na função, bem como ao estresse ocupacional, ao hábito de levar tarefas para casa e à atuação anterior em IES.

Verificou-se que a necessidade de realizar parte do trabalho acadêmico em casa foi fator associado à percepção da pressão por publicação, o que pode se justificar pelo volume de tarefas aliado aos prazos exíguos. O resultado dessa fusão é o aumento no ritmo de trabalho (autoaceleração), que obriga o docente a reorganizar seu tempo, subtraindo horas de descanso. Essa intensificação, embora naturalizada, tem sido associada a consequências físicas e sociais preditoras de estresse crônico^26^. Consoantes a esses achados estão os estudos de Mendonça-Lima e Lima Filho^27^, que apontam a sobrecarga de trabalho como responsável pela atividade docente nos fins de semana e em períodos de interrupção do ano letivo.

Trabalho anterior em IES também foi associado à percepção da pressão por publicação nesta pesquisa. Uma hipótese para explicar esse achado concerne às diferenças da organização do trabalho entre IES particulares e públicas, (78,5% dos docentes haviam trabalhado em IES particular). Nas IES públicas, a produção bibliográfica docente é maior que em IES particulares^28^. É possível que, na mudança de instituição, a cultura produtivista resulte em sobrecarga de trabalho.

Não encontramos nesta pesquisa associação estatisticamente significante entre a percepção da pressão por publicação e a insatisfação no trabalho. Possivelmente a gratificação na carreira docente supere a percepção negativa da cobrança por metas, posto o caráter impessoal e não imediatamente “interessado” do trabalho, tornando possível a autorrealização^29^. Nesse contexto há ainda que se considerar o baixo número da amostra.

Vale destacar que o recorte transversal do desenho do estudo não permite estabelecer relação causal das associações entre os fatores psicossociais do trabalho e a percepção da pressão por publicação. Por se tratar de amostra não probabilística, é possível que tenha ocorrido viés de participação, em que os docentes com maiores percepções de cobrança por demandas de trabalho tenham tido maior interesse em participar da pesquisa. Por fim, a baixa taxa de adesão (aproximadamente 37% da população) pode ser explicada pelo período em que a pesquisa foi realizada (outubro a dezembro de 2018). Segundo justificativa dos próprios docentes, o final do ano é a época em que o volume de trabalho aumenta, e os prazos expiram.

Os achados desta pesquisa sugerem que a organização do trabalho e a saúde mental dos trabalhadores estão inter-relacionados, chamando atenção para a gestão de ambientes universitários, a qual não tem sido suficientemente considerada em estudos sobre saúde mental no trabalho. É importante destacar que, embora em todas as análises o estresse tenha sido considerado como variável dependente, não é possível afirmar categoricamente a direção da associação.

Em resumo, quanto maior a percepção de pressão por publicação, maior o estresse no trabalho. No entanto, esse resultado parece não se refletir na satisfação ou insatisfação do trabalho. O prolongamento das horas de trabalho é aparentemente deliberado pelo docente, mas oculta a precarização e intensificação do trabalho a que os professores, tanto de universidades quanto do ensino básico, têm sido submetidos nos últimos anos pelas políticas públicas que mercantilizam a educação no Brasil.
